# Diverse approaches to predicting drug-induced liver injury using gene-expression profiles

**DOI:** 10.1186/s13062-019-0257-6

**Published:** 2020-01-15

**Authors:** G. Rex Sumsion, Michael S. Bradshaw, Jeremy T. Beales, Emi Ford, Griffin R. G. Caryotakis, Daniel J. Garrett, Emily D. LeBaron, Ifeanyichukwu O. Nwosu, Stephen R. Piccolo

**Affiliations:** Department of Biology, Brigham Young University, Provo, UT USA

**Keywords:** Machine learning, Classification, Cell lines, Drug development, Precision medicine

## Abstract

**Background:**

Drug-induced liver injury (DILI) is a serious concern during drug development and the treatment of human disease. The ability to accurately predict DILI risk could yield significant improvements in drug attrition rates during drug development, in drug withdrawal rates, and in treatment outcomes. In this paper, we outline our approach to predicting DILI risk using gene-expression data from Build 02 of the Connectivity Map (CMap) as part of the 2018 Critical Assessment of Massive Data Analysis CMap Drug Safety Challenge.

**Results:**

First, we used seven classification algorithms independently to predict DILI based on gene-expression values for two cell lines. Similar to what other challenge participants observed, none of these algorithms predicted liver injury on a consistent basis with high accuracy. In an attempt to improve accuracy, we aggregated predictions for six of the algorithms (excluding one that had performed exceptionally poorly) using a soft-voting method. This approach also failed to generalize well to the test set. We investigated alternative approaches—including a multi-sample normalization method, dimensionality-reduction techniques, a class-weighting scheme, and expanding the number of hyperparameter combinations used as inputs to the soft-voting method. We met limited success with each of these solutions.

**Conclusions:**

We conclude that alternative methods and/or datasets will be necessary to effectively predict DILI in patients based on RNA expression levels in cell lines.

**Reviewers:**

This article was reviewed by Paweł P Labaj and Aleksandra Gruca (both nominated by David P Kreil).

## Background

Drug-induced liver injury (DILI) is a serious concern during both drug development and the treatment of human disease. DILI is characterized by elevated levels of alanine aminotransferase; in serious cases, it can ultimately result in acute liver failure and patient death [1]. Reactive drug metabolites may play a role in initiating DILI [1]. Drug hepatotoxicity plays an important role in risk-benefit assessment during drug development, but the ability to accurately predict the risk of DILI for a new drug has evaded investigators [2]. Historically, nearly one third of drug withdrawals may have been related to hepatotoxicity [3]. The ability to accurately predict DILI risk could yield considerable reductions in drug-attrition and drug-withdrawal rates as well as improved treatment outcomes [4].

The 2018 Critical Assessment of Massive Data Analysis (CAMDA) Connectivity Map (CMap) Drug Safety Challenge was held in conjunction with the Intelligent Systems for Molecular Biology conference in Chicago, Illinois. The challenge organizers instructed participants to train predictive models on gene-expression data from Build 02 of CMap [5]. CMap was created to facilitate the discovery of connections among drugs, genes, and human diseases [6]. CMap contains gene-expression profiles from cell lines that were systematically exposed to a range of bioactive small molecules [5]. For the CAMDA challenge, the class labels were binary values indicating whether treatment with a given drug was associated with liver injury in cell-based screens for the following cell lines: MCF7 (breast cancer) and PC3 (prostate cancer). Per the terms of the CAMDA challenge, we used data for 190 small molecules (of the 1309 total small molecules available in CMap) during model training and 86 additional small molecules for model testing. During Phase I of the challenge, the organizers asked each team to submit DILI predictions for the test set. Later the class labels were revealed to the challenge participants to enable follow-up analyses in Phase II.

**Fig. 1:**
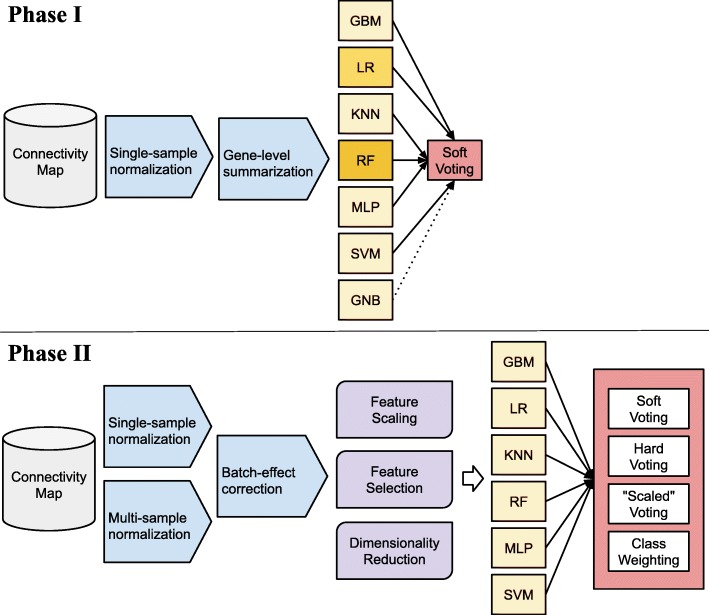
Workflow diagram illustrating analysis approach. In Phase I, we used a single-sample normalization method and gene-level summarization to preprocess the data. Via cross validation on the training set, we evaluated 7 classification algorithms and a soft-voting based ensemble classifier. After receiving class labels for the test set, we performed additional analyses in Phase II. These included using a multi-sample normalization method, batch-effect correction, feature scaling, feature selection, and dimensionality reduction. We also evaluated “hard” voting (treating individual predictions as discrete values), “scaled” voting (using predictions for multiple hyperparameter combinations as input to the voting classifiers), and class weighting (assigning a higher or lower weight to each class label). GBM = Gradient Boosting Machines; LR = Logistic Regression; KNN = K-nearest Neighbors; RF = Random Forests; MLP = Multilayer Perceptron; SVM = Support Vector Machines; GNB = Gaussian Naïve Bayes

In Phase I, we evaluated seven classification algorithms on the training data (Fig. 1). In addition, we used a soft-voting classifier, which combined the outputs of the individual classifiers. This technique often outperforms individual classifiers that are used as input to a voting ensemble [7]. Generally, voting-based approaches are most effective when they incorporate individual classifiers that perform reasonably well in isolation and when the component classifiers use diverse methodological approaches and thus are more likely to have deficiencies in different areas of the input space, often allowing for improved performance in aggregate [8, 9]. We hoped that this would hold true for predicting DILI in this study because the individual algorithms that we used represent diverse methodological approaches.

After submitting our predictions to the challenge organizers, we learned that our predictions performed worse than random-chance expectations. Thus, during the second phase of the challenge, we explored various options for improving classification accuracy, including different preprocessing methods, feature-selection and feature-transformation approaches, class weighting, and multiple hyperparameter combinations (Fig. 1).

## Results

### Phase I

**Fig. 2:**
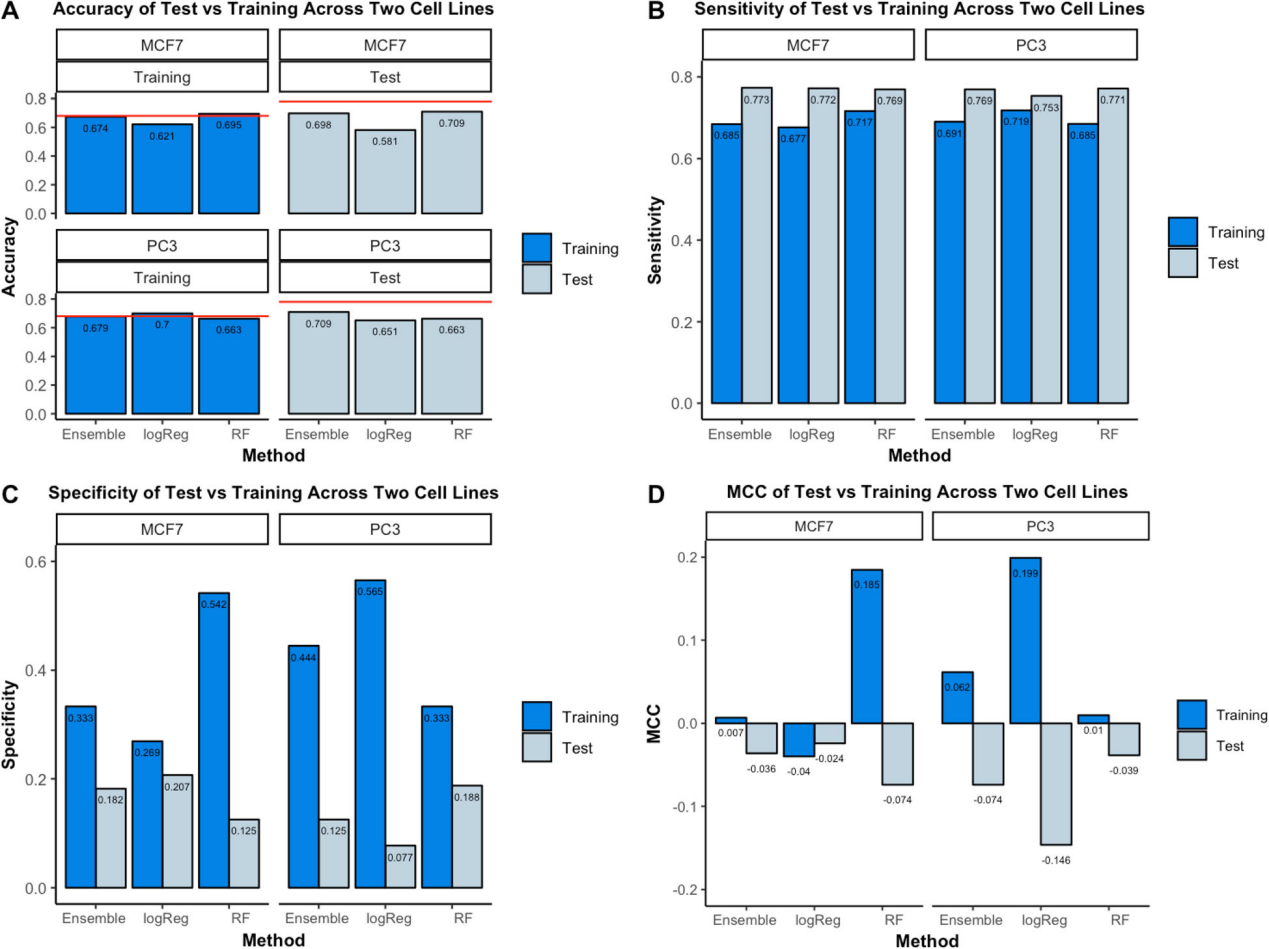
Phase I training and test results of our three submitted classifiers. Using the training data, we evaluated and attempted to optimize 7 classification algorithms as well as a soft-voting based classifier. Based on this analysis, we selected three approaches: soft voting (Ensemble), a Logistic Regression classifier (logReg), and a Random Forests classifier (RF). After evaluating these predictions, the CAMDA Challenge organizers provided class labels for the test set. These graphs illustrate the performance of the classifiers on the training and test sets during Phase I. **a** In some cases, the classifiers outperformed baseline accuracy (red lines), which reflect the predictive performance when classifying all cell lines as the majority class. However, the classifiers performed only marginally better—and sometimes worse—than the baseline. **b-c** Sensitivity increased and specificity decreased for the test-set predictions relative to the training-set predictions; this reflects different levels of class imbalance between the training and test sets. **d** On the training set, the Matthews Correlation Coefficient (MCC) was sometimes better than expected under random-chance expectations, but it was always worse on the test set

During Phase I, we used cross validation to evaluate seven classification algorithms, multiple hyperparameter combinations for each of these algorithms, and a voting-based classifier that aggregated these individual classifiers. Table 1 summarizes the hyperparameter values used in our final solutions. The CAMDA challenge allowed us to submit three solutions. Based on our cross-validation results, we selected the following algorithms: 1) Logistic Regression, 2) Random Forests, and 3) the soft-voting method. We trained these algorithms on the full training set, made predictions on the test set (before seeing the true class labels for these samples), and then submitted our predictions to the CAMDA challenge organizers. We chose the Logistic Regression and Random Forests classifiers because they resulted in relatively high MCC values (Table 2). We chose the voting-based classifier because of its consistent performance across all metrics (Table 2). Although the voting method’s performance was slightly lower than the best individual algorithms on the training data, we anticipated that it would be relatively effective on the test data because it would be robust to poor performance of individual algorithms while benefiting from a diversity of predictions. After Phase I concluded, we received a report indicating the performance of our solutions on the test set (Fig. 2). We also received class labels for the test set so we could evaluate additional alternatives for optimizing predictive performance.

**Table 1:** Summary of classification algorithms evaluated on the training set

Classification algorithm	scikit-learn implementation	Parameters selected after optimization
Multilayer Perceptron	sklearn.neural_network.MLPClassifier	activation = ‘relu’ alpha = 0.0001 batch_size = ‘auto’ beta_1 = 0.9 beta_2 = 0.999 early_stopping = False epsilon = 1e-08 **hidden_layer_sizes = (30,30,30,30,30,30,30,30,30,30)** learning_rate = ‘constant’ **learning_rate_init = 0.0376** max_iter = 200 momentum = 0.9 nesterovs_momentum = True power_t = 0.5 random_state = None shuffle = True solver = ‘adam’ tol = 0.0001 validation_fraction = 0.1 warm_start = False
Gradient Boosting	sklearn.ensemble. GradientBoostingClassifier	criterion = ‘friedman_mse’ init = None **learning_rate = 0.31** loss = ‘deviance max_depth = 3 max_features = None max_leaf_nodes = None min_impurity_decrease = 0.0 min_impurity_split = None min_samples_leaf = 1 min_samples_split = 2 min_weight_fraction_leaf = 0.0 n_estimators = 100 presort = ‘auto’ subsample = 1.0 warm_start = False
K-nearest Neighbor	sklearn.neighbors.KNeighborsClassifier	algorithm = ‘auto’ leaf_size = 30 metric = ‘minkowski’ metric_params = None **n_neighbors = 8** *p* = 2 **weights = ‘distance’**
Logistic Regression	sklearn.linear_model.LogisticRegression	C = 1.0 class_weight = None dual = False fit_intercept = True intercept_scaling = 1 max_iter = 100 multi_class = ‘ovr’ penalty = ‘l2’ **solver = ‘lbfgs’** tol = 0.0001 warm_start = False
Gaussian Naïve Bayes	sklearn.naive_bayes.GaussianNB	priors = None
Random Forest	sklearn.ensemble. RandomForestClassifier	**bootstrap = False** class_weight = None criterion = ‘gini’ **max_depth = 9** min_samples_split = 2 min_samples_leaf = 1 min_weight_fraction_leaf = 0.0 max_features = ‘auto’ **max_leaf_nodes = 25** min_impurity_decrease = 0.0 min_impurity_split = None **n_estimators = 25** oob_score = False warm_start = False
Support Vector Machines	sklearn.svm. SVC	C = 1.0 class_weight = None coef0 = 0.0 decision_function_shape = ‘ovr’ degree = 3 gamma = ‘auto’ kernel = ‘rbf’ max_iter = − 1 probability = False shrinking = True tol = 0.001
Voting-based Ensemble	sklearn.ensemble. VotingClassifier	flatten_transform = True **voting = ‘soft’** weights = ‘None’

In Phase I, we employed 7 classification algorithms and a voting-based method that integrated predictions from the individual classifiers. The first two columns indicate a name for each algorithm and the scikit-learn implementation that we used for each algorithm. Using an ad hoc approach, we evaluated many hyperparameters via cross validation on the training set and selected a hyperparameter combination for each algorithm that performed best. Non-default parameters are bolded. Hyperparameters that do not fundamentally affect algorithm behavior—such as the number of parallel jobs—are not shown

**Table 2:** Phase I cross-validation results

	Accuracy		Sensitivity		Specificity		MCC	
	*PC3*	*MCF7*	*PC3*	*MCF7*	*PC3*	*MCF7*	*PC3*	*MCF7*
Multilayer Perceptron	0.63	0.65	0.69	0.69	0.32	0.35	0.01	0.03
Gradient Boosting	0.67	0.60	0.69	0.67	0.39	0.27	0.04	−0.05
K-nearest Neighbor	0.68	0.64	0.70	0.72	0.50	0.41	0.11	0.12
Logistic Regression	**0.70**	0.62	0.72	0.68	**0.57**	0.27	**0.20**	−0.04
Gaussian Naïve Bayes	0.35	0.35	0.71	0.73	0.32	0.32	0.02	0.03
Random Forest	0.66	**0.70**	0.69	0.72	0.33	**0.54**	0.01	**0.19**
Support Vector Machines	0.68	0.68	**1.00**	**1.00**	–	–	–	–
Voting-based Ensemble	0.68	0.67	0.69	0.69	0.44	0.33	0.06	0.01

These results indicate how each classification algorithm performed on the training set after hyperparameter tuning. Overall, the Logistic Regression and Random Forests algorithms performed best,thus we selected these for submission to the challenge. The voting-based ensemble never outperformed all the individual algorithms, yet it never performed worse than all the individual algorithms. Thus we also constructed a submission for the challenge based on this classifier. PC3 and MCF7 are names of prostate- and breast-cancer cell lines, respectively. Bolded values indicate relative strong performance for the three algorithms we selected in Phase I. MCC = Matthews Correlation Coefficient. We were unable to calculate specificity or MCC for the Support Vector Machines algorithm because it predicted all cell lines to have the same class label

**Fig. 3:**
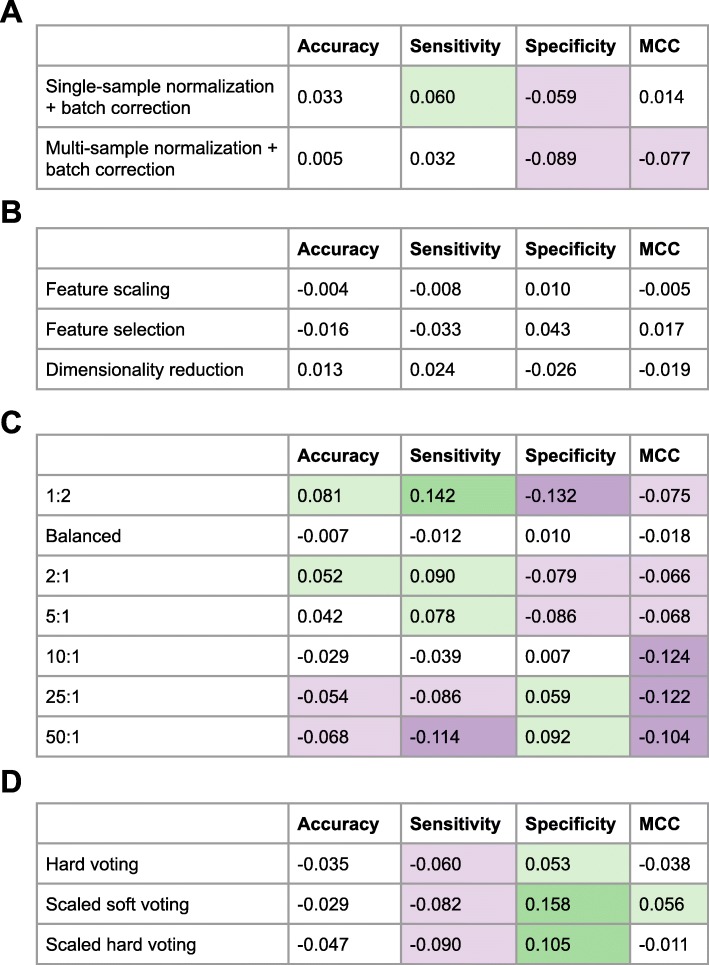
Relative gain (or loss) in classification performance after Phase II optimizations, relative to Phase I. In Phase II, we implemented 4 types of changes to our classification approach in an attempt to improve performance relative to Phase I. For each type of adjustment, the numbers in this figure represent average differences across all relevant classification algorithms. (The *class_weight* hyperparameter only applies to some classification algorithms; we calculated averages only for the algorithms that supported it). Green indicates relatively high performance compared to Phase I on the test set; purple indicates lower performance. **a** Performance metrics for data that had been normalized using either the SCAN or FARMS algorithm after batch adjustment with Combat. **b** Performance metrics after each variable had been scaled, after feature selection, or after dimensionality reduction. **c** Performance metrics after altering weights assigned to each class label. Numbers indicate weights assigned to the non-DILI vs. DILI class labels. **d** Performance metrics for variations on the voting-based ensemble approach. The hard-voting approach combined binarized predictions across the algorithms, whereas soft voting used probabilistic predictions. The scaled methods combined predictions from default and non-default hyperparameter combinations for each algorithm

In Phase I, none of our solutions produced consistently accurate predictions on the test set (Fig. 2). Accuracy for the voting-based classifier increased relative to its performance on the training dataset, but it was well below baseline accuracy (predicting the majority class by default). Our classifiers appeared to be unable to effectively account for the imbalance between hepatotoxic and non-hepatotoxic drugs in the CMap dataset. Our classifiers tended to predict hepatotoxic vs. non-hepatotoxic outcomes in proportions that reflected the training dataset. However, the test set included fewer molecules that were hepatotoxic than the training set; thus our models predicted hepatotoxic outcomes too frequently. This is reflected in the performance metrics for the test dataset, in which our models achieved increased sensitivity but decreased specificity (Fig. 3b-c).

### Phase II

In addition to providing class labels for the test set, the CAMDA organizers provided us with suggestions from reviewers. These suggestions gave us ideas for improving classification performance, which we evaluated in Phase II. Because we did not have an additional, independent dataset, our Phase II evaluations were only exploratory in nature. We explored four types of techniques for improving performance: a multi-sample normalization method and batch correction, feature scaling/selection/reduction techniques, custom class weights, and scaling of the voting-based ensemble method. To quantify the effects of these alternative approaches, we compared the performance of our classifiers with and without each change, averaged across all classification algorithms—with the exception of adjusting the class weights, which was only possible for a subset of the algorithms (see Methods). Figure 3 illustrates the effects of these changes.

In Phase I, we preprocessed the microarray array using the SCAN algorithm, a single-sample normalization method. We hypothesized that preprocessing the data using the FARMS algorithm (a multi-sample normalization method) would result in improved performance by reducing technical variability across the samples via quantile normalization. In addition, because the CMap data had been processed in many batches, we hypothesized that correcting for batch effects using the ComBat algorithm would increase classification performance. In some cases, these changes improved predictive performance slightly, whereas in other cases the performance was reduced, irrespective of whether we used SCAN, FARMS, and/or batch adjustment (Fig. 3a).

Although microarray normalization methods help to remove technical biases and multi-sample corrections can remove inter-sample variations, some classification algorithms assume that each feature has been scaled to have the same mean and standard deviation. Accordingly, in Phase II, we used scikit-learn’s RobustScaler functionality to scale the expression data for each gene; this method also adjusts for any outliers that may exist. Secondly, we reduced the feature space via feature selection (using the ANOVA F-value) and dimensionality reduction (using Principal Component Analysis). These adjustments did not improve performance consistently (Fig. 3b).

In an attempt to mitigate the effects of class imbalance, we adjusted weights assigned to the class labels. By default, classification algorithms in scikit-learn place an equal weight on each class label, but many algorithms provide an option to adjust these weights. We attempted many different weight ratios, even placing 50 times more weight on the minority class than the majority class. These adjustments often improved sensitivity or specificity, but none of these changes resulted in a higher MCC value (Fig. 3c).

Finally, we made various attempts at improving the voting-based classifier. We used hard voting rather than soft voting. With this approach, the predictions for the individual classifiers are treated as discrete rather than probabilistic values, which may improve ensemble predictions in situations where probabilistic predictions are poorly calibrated. In addition, we increased the number of individual classifiers used for voting. We retained the same classification algorithms, but we included predictions for multiple hyperparameter combinations per algorithm. We suspected that a larger and more diverse set of predictions would improve voting performance. None of these approaches resulted in consistent improvements for any of the metrics except specificity (Fig. 3d); these were counterbalanced by decreases in the other metrics.

## Discussion

Our goal was to make progress toward accurately predicting DILI based on gene-expression profiles of cell lines. The ability to predict these outcomes could reduce patient injury, lower costs associated with drug development, and optimize treatment selection. As a step toward these objectives, we analyzed gene-expression levels from cancer cell lines that had been treated with small molecules; we used machine-learning classification to predict DILI. Our study design relied on the assumption that drugs causing liver injury induce transcriptional changes that are common across many or all of these drugs and that these transcriptional changes might also occur in liver tissue in vivo.

In Phase I, we employed seven classification algorithms as well as a soft-voting ensemble classifier that aggregated predictions from six of the seven individual algorithms. On the training data, we observed relatively high performance for the Random Forests and Logistic Regression algorithms, which coincides to an extent with prior findings [10]. However, when applied to the test set, neither algorithm consistently produced predictions that exceed what can be attained by defaulting to the majority class. The soft-voting approach yielded better performance than the individual algorithms at times, but this pattern was inconsistent. Voting-based approaches often outperform single-classifier approaches because they combine diverse algorithmic techniques—where one algorithm fails, other(s) may succeed. However, they rely on a diverse range of inputs; using algorithms from a narrow range of methodologies will generally be less performant.

We emphasize the importance of considering multiple, diverse performance metrics when evaluating classification results. Even though our classification algorithms sometimes attained higher levels of accuracy on the test set than the training set (Fig. 2a), these improvements were likely a consequence of different levels of class imbalance between the training and test sets—a higher proportion of drug compounds induced liver injury in the training samples than in the test samples. Our classifiers were prone to over-predicting liver injury. Although accuracy and sensitivity typically benefitted from this bias, specificity typically offset these gains when considered in the broader context. Accordingly, we believe that the degree of class imbalance was a key reason that our methods underperformed. To address this limitation in Phase II, we assigned higher weights to the minority class, thus potentially helping to account for class imbalance. Even though this approach rests on a solid theoretical foundation [11], it resulted in minimal, if any, improvements in overall performance.

Additionally, we attempted to improve classification performance using a multi-sample normalization method, adjusting for batch effects, scaling features, selecting features, reducing data dimensionality, and using multiple hyperparameter combinations as input to the voting-based classifier. Although these techniques might have resulted in improvements in other classification scenarios, they resulted in minimal improvements, if any, in predictive ability in our analysis. The batch-effect correction method that we used (ComBat) requires the researcher to assign batch labels to each biological sample. Alternative tools such as PEER [12] and SVA [13] can be used in situations where batch labels are unknown or more generally to detect hidden variation. Indeed, hidden factors—perhaps due to treatment duration and physiological complexity—–may have confounded this study. DILI was determined based on a meta-analysis of patient data, whereas our predictions were derived from treatments administered to cell lines over the course of only a few hours or days.

## Conclusions

The original goal of this CAMDA challenge was to predict hepatic injury from mRNA expression profiles. Our findings suggest that some or all of the following factors may explain our limited success in predicting these outcomes: 1) gene-expression microarray measurements are often noisy, 2) mRNA expression levels in cell lines may be inadequate surrogates for in vivo responses in this setting, 3) larger datasets may be needed, and 4) more sophisticated analytic techniques may be needed.

## Methods

### Data preprocessing

The training set was a subset of CMap consisting of gene-expression data and known DILI status for 190 small molecules (130 of which had been found to cause DILI in patients). The test set consisted of an additional 86 small molecules. The CMap gene-expression data were generated using Affymetrix gene-expression microarrays. In Phase I, we used the Single Channel Array Normalization (SCAN) algorithm [14]—a single-sample normalization method—to process the individual CEL files (raw data), which we downloaded from the CMap website (https://portals.broadinstitute.org/cmap/). As part of the normalization process, we used BrainArray annotations to discard faulty probes and to summarize the values at the gene level (using Entrez Gene identifiers) [15]. We wrote custom Python scripts (https://python.org) to summarize the data and execute analytical steps. The scripts we used to normalize and prepare the data can be found here: https://osf.io/v3qyg/.

For each treatment on each cell line, CMap provides gene-expression data for multiple biological replicates of vehicle-treated cells. For simplicity, we averaged gene-expression values across the multiple vehicle files. We then subtracted these values from the corresponding gene expression values for the compounds of interest. Finally, we merged the vehicle-adjusted data into separate files for MCF7 and PC3, respectively.

The SCAN algorithm is designed for precision-medicine workflows in which biological samples may arrive serially and thus may need to be processed one sample at a time [14]. This approach provides logistical advantages and ensures that the data distribution of each sample is similar, but it does not attempt to adjust for systematic differences that may be observed across samples. Therefore, during Phase II, we generated an alternative version of the data, which we normalized using the FARMS algorithm [16]—a multi-sample normalization method. This enabled us to evaluate whether the single-sample nature of the SCAN algorithm may have negatively affected classification accuracy in Phase I. Irrespective of normalization method, it is possible that batch effects can bias a machine-learning analysis. Indeed, the CMap data were processed in many batches. Therefore, for SCAN and FARMS, we created an additional version of the expression data by adjusting for batch effects using the ComBat algorithm [17].

### Feature selection

Initially in Phase I, we used a variance-based approach for feature selection (with the goal of identifying which genes would be most informative for classification). We calculated the variance of the expression values for each gene across all samples; then we selected different quantities of genes that had the highest variance and used those as inputs to classification. However, in performing 10-fold cross validation on the training set, we observed no improvement in classification performance regardless of the number of high-variance genes that we used, so we decided not to use feature selection for our Phase I predictions. To perform cross-validation, we wrote custom Python code that utilizes the scikit-learn module (version 0.19.2), [18].

In Phase II, we used the following scaling and feature-selection methods in an attempt to improve performance: robust scaling, feature selection based on the ANOVA F-value, and principal component analysis. We used scikit-learn implementations of these methods and used default hyperparameters [18].

### Classification

We performed classification using the following algorithms from the scikit-learn library: Gradient Boosting [19], Logistic Regression [20], K-nearest Neighbors [21], Random Forests [22], Multilayer Perceptron [23], Support Vector Machines [24], and Gaussian Naïve Bayes [25]. For each of these algorithms, we used scikit-learn to generate probabilistic predictions. For the voting-based ensemble classifier, we used the VotingClassifier class in scikit-learn. In Phase I, we used “soft” voting, which averages probabilistic predictions across the individual classifiers [8]. In Phase II, we used “hard” voting, which predicts the class label as that which received the larger number of discrete votes.

**Fig. 4:**
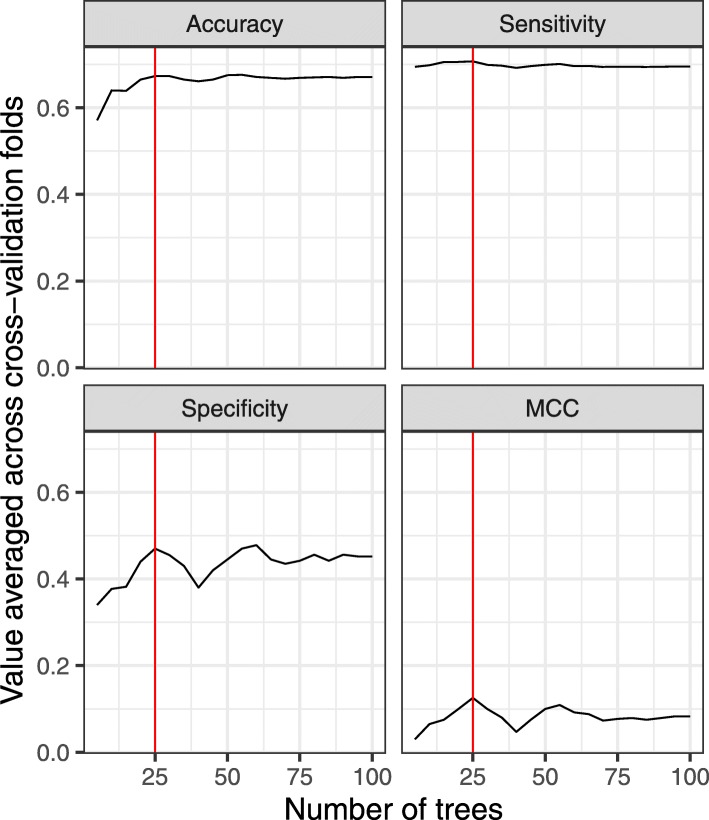
Phase I results of hyperparameter optimization based on the “number of trees” hyperparameter for the Random Forests algorithm. We used an ad hoc approach to tune algorithm hyperparameters on the training set. As an example, we tuned the “number of trees” hyperparameter for the Random Forests algorithm. The performance varied considerably for different numbers of trees. All 4 metrics peaked near 25 trees (red lines). MCC = Matthews correlation coefficient

In Phase I, we sought to select optimal hyperparameter values for each algorithm via cross validation on the training set. We evaluated a variety of parameter combinations (combinations of model settings) for each algorithm, assessing each combination based on its effect on accuracy, specificity, sensitivity, and the Matthews Correlation Coefficient (MCC) [26]. Each of these metrics prioritizes different aspects of classification performance; perhaps the most useful is MCC because it takes into account the frequency of each class label and thus is suitable for evaluating a binomial classifier’s performance when the class is imbalanced, as was true with our data. We used an ad hoc approach to optimize hyperparameters, making judgments about algorithm performance based on visualizations; for example, we evaluated the “number of trees” hyperparameter for the Random Forests algorithm, using values ranging between 5 and 100, and observed relatively high performance across all four metrics when 25 trees were used (Fig. 4). We used a similar approach to optimize additional hyperparameters (e.g., tree depth, maximum leaf nodes, minimum number of samples required to split an internal node, minimum samples per leaf for the Random Forests algorithm). We evaluated each hyperparameter in isolation; a limitation of this approach is that it did not account for possible interactions across hyperparameters.

In an attempt to optimize the performance of the voting-based classifier, we devised a weighting scheme, which assigned higher weights to individual algorithms that performed relatively well during cross validation; we also experimented with excluding individual classifiers from the voting-based classifier. The only approach that appeared to have a consistently positive effect on performance was to exclude the Gaussian Naïve Bayes algorithm, which had also performed poorly in isolation. Our final voting-based model in Phase I excluded Gaussian Naïve Bayes and assigned an equal weight to each individual classifier.

In Phase II, we attempted to improve the voting-based classifier in multiple ways. First, rather than selecting a single hyperparameter combination for each algorithm and using those as input to the voting-based classifier, we used multiple hyperparameter combinations for each classification algorithm (except Gaussian Naïve Bayes). For this approach, we incorporated the following classification algorithms (with the number of distinct hyperparameter combinations): Multilayer Perceptron (*n* = 5), Support Vector Machines (*n* = 4), Logistic Regression (*n* = 2), Random Forests (*n* = 5), K-nearest Neighbor (*n* = 5), and Gradient Boosting classifiers (*n* = 3). We also investigated whether assigning weights to each class label would help overcome the effects of class imbalance and improve classification performance. Four of the classifiers from Phase I—Random Forests, Support Vector Machine, Logistic Regression, and the soft-voting ensemble method—support a *class_weight* hyperparameter, which allowed us to apply custom weights to each class label (or to determine the weights algorithmically). Adjusting the *class_weight* hyperparameter required providing a weight for the non-DILI (weight_1) and DILI observations (weight_2), indicated here as weight_1:weight_2. We used class weights of 50:1, 25:1, 10:1, 5:1, 2:1, 1:1, and 1:2.

## Reviewers’ comments

### Reviewer’s report 1

Paweł P Labaj, Jagiellonian University (nominated by David P Kreil, Boku University Vienna).

## Reviewer comments

The manuscript by G. Rex Sumsion et al. presents ‘An Ensemble Approach to Predicting Drug-induced Liver Injury Using Gene-Expression Profiles’. DILI prediction with use of single source of data (like expression microarrays) is extremely challenging what has been presented in the course of CAMDA conferences. Sometimes it is very valuable to obtain information what will not work and why. In this manuscript a multiple approaches has been tested as well as some ‘improvements’ suggested by CAMDA reviewers, but none is providing really good results. The proposed Ensemble approach is a good idea in such cases, however, I would expect better explanation when Ensemble approach might not work (more specific comments in next point).

1. Overall the manuscript is well written, however, reader can loose a track in both methods and results. Better structure complemented with a figure outlining the analysis procedure would improve readability and by this improve the quality of the manuscript.
2. What is missing in the manuscript is deeper description of Ensemble approach with all pros and cons. This approach could be easily tricked if a few used methods have similar bases / are from close families of solution. Here it is not a case but should be pointed out and described. Connected to this is selection of used methods, just saying that these ones are available ‘scikit-learn library’ is not enough.
3. Authors, in one of the improvements, have used ComBat for batch correction, but this will work only for known confounders. It would be interesting to see, or at least, comment the application of solutions which could detect also hidden confounders, like PEER or SVA.
4. Figure presenting the overview of the analysis and all additions should be provided to improve readability. The additional comment to second point is that CMap is created when cell line has been treated with a specific dose, while DILI is based on meta-analysis of real patients data. One could expect that an important factor for DILI is whether the therapy was short time or prolonged as in the other even small toxicity can accumulate and lead to DILI. Of course the necessary data were not provided here, but it could be that therapy type factor could be detected as hidden confounder.

Authors’ response: *We thank the reviewer for taking the time to review our manuscript and for providing these comments.*

1. We have revised the text in the Methods and Results sections to make the manuscript easier to read. We have also revised the sub-section headings to facilitate better organization. In addition, we have added a figure that illustrates our workflow across the two phases of the CAMDA challenge.
2. We modified the wording in the 3rd paragraph of the Introduction section to say the following: “Generally, voting approaches are most effective when they incorporate individual classifiers that perform reasonably well in isolation and when the component classifiers use diverse methodological approaches and thus are more likely to have deficiencies in different areas of the input space, often allowing for improved performance in aggregate. We hoped that this would hold true for predicting DILI in this study because the individual algorithms that we used represent diverse methodological approaches.” We also modified the Discussion section as follows: “The soft-voting approach yielded better performance than the individual algorithms at times, but this pattern was inconsistent. Voting-based approaches often outperform single-classifier approaches because they combine diverse algorithmic techniques—where one algorithm fails, other(s) may succeed. However, they rely on a diverse range of inputs; using algorithms from a narrow range of methodologies will generally be less performant.” In addition, we have provided an expanded table that shows which parameters we used for each algorithm.
3. We added the following statement to the last paragraph of the Discussion section: “The batch-effect correction method that we used (ComBat) requires the researcher to assign batch labels to each biological sample. Alternative tools such as PEER and SVA can be used in situations where batch labels are unknown or more generally to detect other types of hidden variation.”
4. In complement to the previous point, we have modified the Discussion to add the point that the reviewer mentioned: “… hidden factors—perhaps due to treatment duration and physiological complexity—–may have confounded this study. DILI was determined based on a meta-analysis of patient data, whereas our predictions were derived from treatments administered to cell lines over the course of only a few hours or days.”

### Reviewer’s report 2

Aleksandra Gruca, Silesian University of Technology (nominated by David P Kreil, Boku University Vienna).

## Reviewer comments

The authors analysed dataset from CAMDA 2018 DILI contest. The main goal of the contest is to accurately predict DILI risk of particular drug based on cell lines gene expression data. To achieve this, the authors try different parameter settings for data preprocessing and apply seven classification algorithms that are finally combined in an ensemble approach. The presented work is of a limited novelty. In general, data processing workflow is designed correctly and the analytical steps performed by the authors are typical for such kind of problems. I do not find any flaws in the proposed approach, although I also do not see any novelty in it. On the positive side I notice that the authors have tried several different combinations of methods and parameters in searching for the best outcome. However, none of the applied techniques was able to significantly improve performance of the classifiers which may be due to the fact that DILI dataset from CAMDA 2018 contest is very difficult to analyse as it is characterised by a weak signal.

I have the following comments:

1. The analysed dataset in described very briefly in the paper. The paper is a separate piece of scientific work, therefore authors should not assume that the reader is familiar with CAMDA contest and the dataset, and they should provide more detailed description of analysed data. For example: how many drugs were measured, what is the distribution of objects between DILI and non-DILI class.
2. I suggest adding the figure representing proposed workflow. It would also clarify if the preprocessing steps were performed separately or as a single workflow
3. I notice the following sentence (2nd paragraph of page 8 of the manuscript): “Naive Bayes algorithm, which had performed quite poorly in isolation (Fig. 3)”. However, I cannot see any data in Fig. 3 related to this sentence.
4. In the description of Fig. 3 I notice the following statement: “For each adjustment in our procedure, we measured the performance of all the classifiers (with the exception of adjusting the class_weight hyperparameter, which was only available for the classifiers listed above ( …)” . It is not clear what the authors mean by “classifiers listed above”
5. In the Fig. 1 Y—axes for metrics accuracy, sensitivity and specificity are not scaled the same way and are of different ranges. As usually values all of these measures are interpreted with the same range, presenting them on different scales might be misleading. I suggest either put all of them on the same Figure or at least present them on a charts that have the same Y-axis range.

Authors’ response: *We thank the reviewer for taking the time to review our manuscript and for providing these comments.*

1. We now provide information about sample sizes and class imbalance in the Data preprocessing section of Methods.
2. We have added a workflow diagram that illustrates the key components of Phases I and II.
3. We thank the reviewer for catching this. We have removed the part in parenthesis from the manuscript.
4. We have thoroughly revised this figure caption (as well as the others) to improve clarity.
5. We have updated this figure according to the reviewer’s suggestion (using the same Y-axis scale for all 4 sub-figures).

## Data Availability

The CMap website (https://portals.broadinstitute.org/cmap/) contains the raw data used in our analysis. The normalized data used in our analysis—along with code we used for normalization—can be found here: https://osf.io/v3qyg/.
